# Myth, Power and Practice: A Bourdieusian Interpretation of Greentown’s Criminal Network

**DOI:** 10.3390/bs16061012

**Published:** 2026-06-17

**Authors:** Andy Bray, Séan Redmond

**Affiliations:** 1 Centre for Implementation Research, University of Limerick, V94 T9PX Limerick, Ireland; 2 Research Evidence into Policy, Programmes and Practice, University of Limerick, V94 T9PX Limerick, Ireland; sean.redmond@ul.ie

**Keywords:** organised crime networks, youth criminal involvement, Bourdieu’s Theory of Practice, habitus, field and capital, symbolic power, symbolic violence, criminal network governance, social capital in criminal networks, making and criminal authority, Greentown case study

## Abstract

This paper offers a theoretical reinterpretation of the groundbreaking Greentown study using Pierre Bourdieu’s Theory of Practice. Rather than presenting new empirical findings, it examines previously published research to study children’s involvement in organised crime networks through a relational, practice-based lens. Dominant approaches to youth offending and gang participation tend to focus on individual risk factors, programme effectiveness or structural indicators and can struggle to account for the enduring social logics through which criminal authority is reproduced across generations. Drawing on Bourdieusian concepts of field, capital and symbolic power, the paper interprets Greentown as a localised social field in which a core family network accumulates and deploys social, cultural, economic and symbolic capital to secure compliance, cultivate loyalty and sustain informal forms of governance. Attention is paid to the role of symbolic narratives and mythmaking in minimising the visible presence of the state and normalising participation for young people and residents. The analysis illustrates how such symbolic orders can persist even where individual agents desist, contributing to the relative stability of networked harm. The paper argues that Bourdieu provides a coherent and theoretically disciplined framework for understanding organised criminal networks as socially embedded fields and suggests that interventions attentive to symbolic power and misrecognition may complement existing criminal justice responses. While explicitly interpretive in scope, the paper points towards the value of theory-led re-readings of empirical research for addressing the complex and ‘wicked’ nature of organised networked offending.

## 1. Introduction

Crime networks present enduring and complex challenges for societies, frequently operating in ways that undermine or displace legitimate governance and produce forms of criminalised, conscious opposition to the state (Sparrow, 2008, pp. 199–216). In some settings, these dynamics give rise to what Sparrow describes as resistant and malign equilibria, in which criminal authority becomes normalised and difficult to disrupt (Sparrow, 2008, pp. 231–241). The involvement of children within such networks, as both participants and social subjects, adds a further layer of complexity to what are often characterised as ‘wicked’ problems (Rittel & Webber, 1973). Children’s participation challenges conventional policy and practice frameworks by straddling the uneasy boundary between law enforcement and child protection, exposing the limitations of approaches that treat these domains as conceptually or institutionally distinct.

This interpretive paper contributes to this Special Issue by arguing that many of the difficulties encountered in addressing children’s involvement in organised crime networks are not solely technical or programmatic, but conceptual. Existing literatures tend to emphasise measurable risk factors, intervention outcomes or structural features of networks (Sparrow, 2008, pp. 85–86), while parallel debates focus on the variable effectiveness of evidence-based programmes when applied to complex, multi-systemic social problems (Mulcahy et al., 2024; C. M. Naughton et al., 2023). While pragmatic analytical tools such as social network analysis have proven valuable in mapping relational structures and informing practice, these mid-level approaches often remain limited in their capacity to account for the deeper social logics through which criminal authority, legitimacy and compliance are produced and reproduced. As Wacquant (2014, pp. 8–9) observes, such accounts risk overlooking the structured practices and symbolic orders that endow certain crime networks with institutional-like properties and durability over time.

This paper seeks to add conceptual value to existing technical understandings of crime networks and children’s involvement by applying high-level social theory, specifically Bourdieu’s Theory of Practice, as an interpretive lens through which to reconsider how crime may be reproduced through dispositions and resources within a social space. Rather than advancing prescriptive solutions, the paper suggests that a Bourdieusian perspective can offer a way of rethinking how state interventions engage with the embedded social logics that appear to shape, legitimise and sustain certain organised criminal networks. Bourdieu’s Theory of Practice is particularly useful in this regard because it foregrounds the social and relational forces that structure young people’s involvement in criminal network activity, dimensions that are often under-theorised within more mechanistic or programme-focused accounts of crime networks and standardised evidence-based intervention models. The analysis takes the Greentown study as an empirical point of reference, drawing on previously published material to illustrate how Bourdieusian concepts can be used to reinterpret the reproduction of organised crime within a localised social field.

The paper unfolds as follows. First, it introduces Bourdieu’s theoretical framework, outlining the concepts of field, habitus and capital, and explaining their relevance for understanding the development of social practice. Second, it provides a concise account of Greentown, the empirical context for the analysis. The Greentown study was originally published in Ireland in 2016, with replications of the study (Bluetown and Redtown) published in 2021 and intervention design contributions informed by the study in 2023 and 2024. The paper then operationalises and applies Bourdieusian concepts to the Greentown material. Drawing on this reinterpretation, the discussion reflects on the value added by Bourdieu’s Theory of Practice in framing the problem of children’s involvement in organised crime networks, highlighting limitations in the extant literature. Finally, in Busting Myths, the paper proposes fresh ways of thinking about how taken-for-granted forms of network power might be challenged.

## 2. Bourdieu’s Theory of Practice: Habitus, Field and Capital

Bourdieu’s Theory of Practice offers a relational account of social life that rejects the dichotomy between structure and agency, positing instead that social practices arise from the interplay of habitus, field and capital (Bourdieu, 1977; Bourdieu & Wacquant, 1992). Rather than privileging either determinism or autonomy, Bourdieu situates agency as conditional, formed and channelled by historically sedimented dispositions, the configuration of social arenas and the resources at stake within them. This provides a coherent lens for analysing how dominant groups set discourse and practice, and why these often reproduce existing hierarchies in domains such as youth justice (Wacquant, 2016; Swartz, 1997).

### 2.1. Habitus

Habitus refers to systems of durable, transposable dispositions that orient perception and action without rule following or explicit calculation (Bourdieu, 1990, p. 53). Dispositions are acquired through socialisation, embodied over time and operate across conscious and non-conscious registers. They supply a practical sense of how to be or feel for the game that equips agents to navigate social situations (Bourdieu & Wacquant, 1992). Habitus is historically shaped yet malleable; primary habitus takes root early in life whilst secondary habitus layers are formed through education, work and movement across social spaces—a multi-layered formation (Wacquant, 2014, p. 7; Bourdieu, 1984). At times, this formation can be fragmented or contested, especially during periods of rapid mobility that generate hysteresis, in which dispositions shaped in earlier conditions no longer align with the transformed logic of the field (Bourdieu, 2000; Wacquant, 2014; Friedman, 2016).

A frequent critique of Bourdieu is that his theory risks determinism, with habitus seen as overly constraining. However, Bourdieu (1977, 1990) and Bourdieu and Wacquant (1992) explicitly reject this interpretation, emphasising that habitus is neither fixed nor automatically determining. Rather than acting as a rigid blueprint, habitus functions as a system of dispositions, or flexible guiderails, that generate practices while remaining open to revision as agents encounter new conditions (Bourdieu, 2000, p. 161). Habitus enables improvisation and adaptation rather than dictating behaviour, and it only operates in relation to the shifting configurations of field and capital (Swartz, 1997, pp. 104–105). By theorising practice as emerging from this relational interplay, Bourdieu avoids both structural determinism and voluntarism, offering an account in which action is shaped by history yet still allows for reflexivity, change and transformation (Bourdieu & Wacquant, 1992; Sandberg, 2008).

### 2.2. Field

Field names the structured social arenas in which agents vie for position, power and recognition. Fields are relatively autonomous microcosms such as law, art, academia or justice, each containing its own stakes, rules and forms of competence. Positions in a field depend on the volume and composition of capital that agents command. Dominant agents generally seek to conserve the status quo while entrants and subordinates contest it (Bourdieu, 1993; Wacquant, 1998; Swartz, 1997). Crucially, practice emerges from the encounter between disposition and position, the correspondence or disjuncture between habitus and field conditions at a given moment (Wacquant, 2008). Furthermore, Wacquant (2018) further notes that fields operate at the same time as physical and symbolic spaces. Physically, they are embedded in material and social conditions that shape agents’ positions and possibilities. Symbolically, they are structured by classifications, meanings and power relations that exert real effects. Applying a topological lens, he shows how symbolic space corresponds with social and physical space, revealing how symbolic structures organise practice and reproduce hierarchy in the face of opportunities and threats (Wacquant, 2018).

### 2.3. Capital

Capital supplies the means of positioning within the field. Beyond economic capital, Bourdieu identifies cultural, social and symbolic capitals (Bourdieu, 1984). Cultural capital exists in objectified artefacts, embodied competencies and institutionalised forms such as qualifications. Social capital exists in networks and obligations. Symbolic capital manifests as prestige and recognition. Capitals convert into each other over time, and their unequal distribution underwrites social stratification and lifestyle hierarchies across the social space (Bourdieu, 1984; Swartz, 1997). Sitting within the above interconnected fields, the field of power regulates conversion rates among capitals shaping which principles of vision and division dominate (Bourdieu, 1994; Loyal, 2016). Among these, symbolic capital is particularly relevant for understanding how authority operates within the field, and it is important to draw a distinction between symbolic capital, symbolic power and symbolic violence. Symbolic capital refers to the accumulation of reputation, recognition and legitimacy within a field. Symbolic power refers to the capacity to mobilise that capital to shape perceptions and regulate expectations. Symbolic violence refers to the process through which such meanings are internalised and misrecognised, such that domination is experienced as natural or taken for granted. These distinctions are used in the analysis below to differentiate between the resources held by agents, the exercise of authority and the processes through which compliance is normalised. Taken together, Bourdieu’s Theory of Practice posits that social practice is generated through a triadic dynamic. Habitus equips agents with embodied schemata through which agents intuitively navigate the social world; fields furnish the stakes, positions and rules of the game. Finally, capital distributes capacities to act and to be recognised. This approach enables researchers to move beyond surface events which may indicate certain deterministic tendencies to the genesis of action, how routines arise, how practice reproduces and when transformations occur (Bourdieu & Wacquant, 1992).

## 3. The Greentown Network

The case under examination is the phenomenon of child recruitment and retention for crime in the ‘Greentown’ crime network in 2010–2011, first published in 2016 and now revisited for this exercise. The Greentown programme of study has been celebrated at the European level for its ‘unparalleled theoretical foundation’ (EUCPN 2020)^1^ and domestically for its impact ‘across multiple domains’ (HEA 2023)^2^. Additionally, the Greentown findings have contributed directly to the passing of bespoke legislation in Ireland to criminalise the adult grooming of children for crime (DOJ 2024)^3^. Greentown offers a *revelatory* case, ‘an opportunity to Observe and analyse a phenomenon previously inaccessible to social science inquiry’ (Yin, 2018, p. 50), made possible by Twinsight (see below). The study has been replicated four times in different locations in Ireland (pseudonymised as Redtown (C. Naughton et al., 2020), Bluetown (O’Meara Daly et al., 2020), Whitetown and Yellowtown).

**Figure 1:**
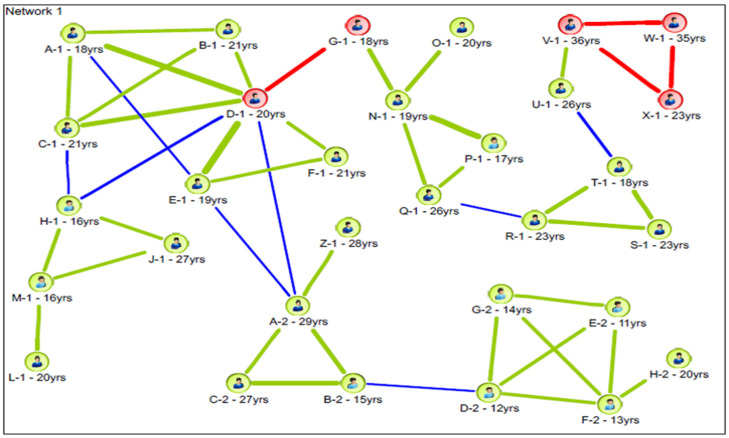
The Greentown network: linkages made using detections of burglary and drugs for sale and supply. Coloured lines reflect the type of criminal activity—Red/drugs, Green/burglary and Blue/other crimes.

Greentown is a real but pseudonymised provincial location in Ireland that was the focus of a PhD study examination (Redmond, 2015). The Greentown crime network was a digitally generated illustration constructed and produced in PDF format by police analysts based on specifications drawn up by the researcher. Minors in the Greentown police district (<18 years) who had been detected for burglary and drugs for sale and supply offences during the period 2010–2011 were identified by analysts along with individuals of any age co-detected with the minors. Each individual on the map is represented as a node (Figure 1 below) along with a unique identifier and specified age (e.g., B-1—21 yrs). Individuals co-detected for the same offences were linked together. This process was repeated and scaled up until the list of minors was exhausted and all relevant links had been made. At this point, the Greentown network was created. The network involved thirty-three individuals, indicating minors and adults involved in burglary and drugs for sale and supply offences. As with any social network, Greentown is a relational presentation of data intentionally bound by category, time and space with inevitable cut points resulting in certain data and parts of the story being excluded.

A detailed qualitative examination of the Greentown network was undertaken using semi-structured interviews with sixteen police officers stationed in Greentown and selected by local police management specially for their significant tacit knowledge of the local area and the individuals involved in Figure 1^4^. A tool designed and developed for the study called *Twinsight*’ (Redmond, 2016) enabled researchers to interview police respondents using the unique identifiers to pinpoint individuals and links between individuals for discussion. Twinsight provided for two versions of the same network. Version A (Figure 1 above) was the shared version of the network map available to both the researcher and the police respondent. However, Version B was provided only to police respondents by the police analyst and was never seen by the researcher. Version B was a direct copy of Version A but augmented with an actual name linked to each node to accompany the unique identifier, referring to a specific individual. In the interview room, the researcher and police officer were positioned sufficiently apart from each to ensure that Version B of the network could not be seen by the researcher and thus remained confidential to the police respondent. The network maps (Version A and Version B) provided the frame of reference for the researcher and police respondent to use, respectively, for discussions about the individuals presented. In summary, Twinsight permitted authentic conversations about the network, simultaneously anonymised for the researcher and case-specific for the police officer. Discussions covered network structures and processes, the nature of relationships and the role of individuals, grounded in people, events and actions to allow for the cross-referencing of the accounts of the police respondents without disclosing the real identities of network members. The individuals identified in this article are directly referred to as letters and numbers, as they are in Figure 1, for example, A1, B1, C1, and D1.

From the semi-structured interviews, the Greentown network emerges for police respondents as a real phenomenon, with real people, real relationships and real narratives. This finding is in itself important for practical research, given that Figure 1 was constructed by analysts who were geographically far away (over 100 km) from Greentown and had no prior knowledge of any of the individuals that they subsequently located in the simulated network. The empirical excerpts drawn on in the analysis that follows are selected purposively from the published Greentown material on the basis of their relevance to the Bourdieusian concepts applied in this paper. Selection was therefore guided by theoretical sensitivity rather than representativeness, with excerpts used illustratively to support this interpretive re-reading.

## 4. Operationalising Bourdieu’s Architecture for Empirical Research

Wacquant (2018) identifies principles that clarify how Bourdieu’s framework can be put to work in empirical research. The first is the need to break with everyday assumptions and construct the research object through deliberate methodological choices. This guards against importing commonsense classifications into analysis and strengthens the precision of concepts used in studying practice. The second principle calls for historicising agents, social worlds and analytic categories, reinforcing Bourdieu’s view that practices emerge from historically situated relations between habitus, field and capital. The third urges researchers to examine how symbolic, social and physical space align with and shape each other, showing how social divisions become embedded in the material organisation of institutions and settings. The fourth principle highlights the formative power of symbolic structures, emphasising that naming and classification shape practice by influencing how agents perceive, value and act within social space. Together, these principles refine the practical use of Bourdieu’s architecture by ensuring that habitus, field and capital are analysed as historically produced, spatially organised and symbolically mediated relations rather than abstract categories applied in isolation. Following Wacquant’s (2018, pp. 8–11) practical guidance that analytical integrity sits better with the application of one concept well than several poorly, we operationalise Bourdieu’s Theory of Practice to focus on the accumulation and conversion of capital by agents as well as the production of ‘myth’ as a governance tool. In the analysis that follows, these concepts are applied as an interpretive framework for re-examining the Greentown material. The network is approached as a field in which agents occupy differentiated positions, habitus is used to interpret patterned dispositions among participants and residents, and different forms of capital are examined in relation to the accumulation and exercise of authority. Particular attention is given to doxa, symbolic power and symbolic violence in order to explore how compliance may be produced, reproduced and normalised within this setting.

## 5. Bourdieu Goes to Greentown

Unless referenced otherwise, the page numbers in this section refer to: Redmond (2015) “Examining the role of criminal networks in causing children to develop longer and more serious crime trajectories” Greentown “a case study.” To maximise transparency, we encourage readers to consult the original PhD study to verify the references used in the sections below and thus judge the plausibility of evidence-informed claims that we make with reference to Bourdieu’s theories.

Greentown is a socially and economically disadvantaged area but with additional properties and characteristics, predisposing those living in Greentown and especially children and adults engaged in its crime network toward compliance with those setting the rules of the game. Children in the network are described as having many of the well-evidenced risks identified as increasing the risk of youth criminality (Farrington & Welsh, 2008), but many have also experienced harsh and abusive child rearing with siblings and parents also involved in criminality (pp. 137–128) and are actively targeted for their vulnerability (p. 139). The network itself is a space of contested sovereignty (p. 128) where the state interventions grapple not only with structural disadvantage, but also with senior network members consciously opposing (Sparrow, 2008, pp. 199–214) attempts to encourage pro-social activity or expose child network members to mainstream norms (p. 155).

Findings in the Greentown study identify an organic hierarchy where an individual agent’s position was determined in large part by whether they were a member of ‘the most feared family in Greentown…’ (p. 126) or not. Agents who had solely offending relationships with the family or leveraged their association with the family to acquire social capital were generally subordinate. Family relationships within the network tended to be governed by ‘trust’ (p. 156) scaffolded by ‘well embedded institutional rules and routines’ (p. 137). Members of the network who did not belong to the core family and kinship group, referred to in the study as associates, were sealed in principal–agent-type relationships with the family (pp. 119–120), characterised by the requirement to comply.

Mechanisms to secure compliance of ‘associates’ included direct violence. Referring to A2 (see Figure 1), deemed to be the elusive head of the family and thus the network, one police respondent noted the following:

*‘I suppose people are so afraid of him. He just has that reputation……... No one would mess with him and he gets that message across in different ways and they know if they mess with him there’s going to be some kind of consequence. And he’s kept that going like he hasn’t left anyone go with things maybe so that’s how he’s keeping his name going and keeping those around him in line as such.’*

*(Interview 009) (p. 98).*

However, A2’s reputation also means that it is not necessary for him to occasion actual harm; he can prompt individuals into action by simply making a phone call (p. 124). It is, however, A2’s subliminal Kurtz-like status (p. 96), the ‘perceptions, stories and myths about A2 and his family’ (p. 123), that is probably the most remarkable and sinister, eliciting reverence toward him by certain young people who covet overt trappings associated with the family (p. 142). The combined violence, threats of violence and stories of violence in the Greentown narrative form a hidden hand to drive compliance with behaviours that are expected (p. 140).

A2 in particular employs certain behavioural characteristics, particularly in his contact with the police. A2 carefully manages his observable activities, requiring ‘associate’ members of the network with lower status to engage in debt collection and meting out violence on his behalf. This feature of A2’s behaviour was highlighted in multiple interviews with police respondents as follows:

*‘If you met him he’s like charming and would talk away to you… (interview 013) (p. 99) ‘You go to A2’s house………. You’ll be invited into the house…….. So you don’t kick the door in or whatever…….. You’d be invited in………… You get in… they open the door they’ll sit with you while you do your search ………….they’ll ask you……..’ I’ll come away with you’………. Everything will be “how are you (Police Officer Christian name)?” “How we’re getting on (Police Officer Christian name)?” “What are you doing today (Police Officer Christian name)?” “What you want me for?... ‘’coming with you? …..no bother, I’ll just get my coat there’’ (Police Officer Christian name) “ ……*

*(Interview 016) (p. 140).*

A2 and the small number of senior members of the network expect similar self-controlled behaviours from other networks members, in particular family members. B2 (aged 15 years at the time of the study) is the brother of A2 and considered the natural heir to control the network. While, at the time of the study, B2 benefited from the trust relationship that characterised the governance of family members, this had only been a recent occurrence because ‘in his early adolescence he was considered impetuous and impulsive’ (p. 100).

Maturation and its relationship with the emergence of trusted status by family members is further evidenced by D2, the son of A2, who at the time of the study was 12 years of age. Police officer respondents reported that D2 is encouraged to commit crime but is also ‘sheltered’ to avoid him inadvertently giving away any of A2 and the network’s intelligence:

*‘As I said to you D2….. Doesn’t be involved as much… Whether it’s a thing that… for all the world …. A2 doesn’t want him involved because he is too young and is a kind of, a liability there…. He may leak something he shouldn’t …*

*(Interview 015) (p. 100).*

The reality for children consciously and intentionally deploying these behavioural practices in all occasions was considered problematic for them. One police officer recounted a situation in which a police interaction with A2’s children required them to be both polite and yet subtly non-compliant. One officer observed (p. 139) the following:

*‘they don’t know what to do because if they don’t talk to us when the parent is there they will be chastised for not having manners, but the minute they go behind closed doors and we are out of ear’s….. We are out of the way of hearing it, they’ll probably being praised for, you know not showing us respect or “don’t give them any information”……. So they don’t know whether they’re coming or going….’*

*(Interview 008).*

Notwithstanding the complexities of self-management, both B2 and D2 carry authority and intimidation rights in Greentown well beyond their actual age and maturity (p. 130). In open community settings, D2’s convivial manner with local police on patrol in the Greentown neighbourhood has an added, sinister governance effect. As one police officer reported, it ‘…..may have the more profound effect of convincing residents that the family has a special relationship (with the police) serving to undermine their confidence in the authorities to alter the status quo’ (p. 130). This is only one example of a wider repertoire of well-understood tactics designed to game the criminal justice system (p. 146).

However, while family members appear at least to try and navigate the complexities of self-governance in a turbulent network setting constantly under threat by state agents, ‘associates’ often do not practice the same discipline, which may be why A2’s governance of these relationships is characterised by compliance as opposed to trust. The numbers of links to F2 and A1 in Figure 1, using basic centrality logic in social network analysis (Morselli, 2010), indicate some degree of seniority in the network. This turns out to be an erroneous assumption. In fact, they are mid- to lower-level agents in the hierarchy. Their disproportionate share of connections (given that the links refer to police detections) means they get caught a lot and are consequently a potential network weakness. F2, along with a group of young people that he has recruited, was involved in a spate of high-profile burglary and robbery offences ‘and appears to represent a chaotic fringe to the Greentown network’. A1 ‘is remembered for hostility to authority, an attitude that appears not to have tempered as he has grown older’ (p. 104). As one police officer recalls:

*‘….You go to A1’s house to arrest him for instance for tax …..or D1…….. The father is coming out of the door drunk abusing you, the brother B1 is probably drunk coming across from the house next door abusing you…. The mother is abusing us all of a sudden…. As you said it’s a fractious atmosphere……. Everybody is erm giving out….*

This distinctive pattern relating to self-management is not exclusive to A1 and F2. D1, who is also an ‘associate’, has some degree of seniority in the network, has saleable skills, not least his driving proficiency, and is an acolyte of A2. D1 generally and deferentially apes A2’s behaviour (p. 122). However, D1 is also capable of poor self-control and had been punished by A2 for dissent, demonstrating that even reasonably senior network actors who are not family members face sanction. They are expected to self-regulate and if not to be kept at a manageable distance.

As one respondent commented, D1 ‘……is not considered to have the right temperament, acumen or intelligence to become leader of the network. Consequently, he is not party to the core intelligence of A2’s network’ (p. 102).

Up to this point, the Greentown network has been characterised by a clear governance dichotomy; relationships based on trust (family) and relationships based on compliance (associates). This is a reasonably accurate portrayal but is insufficient for this examination. Associates appear to possess little or no agency and their relationship with the network is one primarily of being subject to purposeful duress. Nevertheless, however Faustian the deal, the network also represents an opportunity to children and young people, which may be as overt as ‘access to a party lifestyle’ (p. 141) but also relational, deriving ‘patronage, excitement and the acquisition of social capital by association, being seen in the car with A2, D1 or other more senior actors’ (p. 124).

As well as keeping the network in check, A2 and his supporters effect an efficient regime of control over non-involved households in Greentown. As well as functioning as a criminal enterprise, the crime network has extended its activities into moneylending, considered to be a conscious choice by A2 to be involved in less risky activity but also in turn creating client–patron relationships anchored by obligation with poor families (p. 97). Debt to A2 and the network becomes a governance mechanism in itself (p. 119), levering debtors, often women, who had never been involved in crime ‘to become involved in theft and shop-lifting activity to service the debt’ (p. 122). The family and kinship network and its strongest supporters are spatially distributed across the geographical span of Greentown, giving at least the impression to ordinary Greentown residents of ubiquitous surveillance. Most residents want little or no contact with A2 and stoically go about their business, keeping their heads down (p. 129), not wanting to draw attention to themselves; the network’s ‘…organic governance mechanisms [are] seemingly far more influential than any formal agency or threatened criminal court disposal in directing behavior and retaining control….’. Greentown presents as a contested space: ‘…Core network members appear not to be fearful of any sanction for bad behavior. Rather the drift in the system embroiders an untouchable mythology with regard to A2 and his family in the minds of network clients and associates…’ (p. 159). The encroachment of the criminal justice system on this state of criminal equilibrium is limited, resting on ‘shaky presumptions’ (p. 124) of the chances of a subdued population providing witness evidence against A2 and the network. Prison detentions by network members appear to offer only brief respite for the community (p. 204). Importantly, while improving a desistence trajectory is an important objective for the state in relation to the individual offender, it has marginal benefit for the Greentown community if the network, via ongoing recruitment, is capable of sustaining the same volume of criminal activity. Reducing overall network potency as opposed to individual desistance seems more important for residents in Greentown.

## 6. Greentown Goes to Bourdieu

As the discussion proceeds, the Greentown network is approached analytically as a field: a structured social space in which different agents occupy positions and engage in struggles over resources, status and the capacity to shape local norms. Whether Greentown is best understood as a field in its own right or as a sub-field within a wider “street field” (Shammas & Sandberg, 2016) is not a question that can be resolved empirically, nor is it essential to the analytical aims of the paper. Read as a sub-field, Greentown’s hierarchies, opportunities and symbolic valuations can be seen as reflecting broader criminal networks, policing practices and shared meanings of status and risk (Bourdieu, 1984, 2013; Bourdieu & Wacquant, 1992). At the same time, the network exhibits a relatively coherent relational structure, with distinctive forms of capital, struggles for influence and an internal logic that can be usefully interpreted through the notion of a field-specific habitus (Bourdieu, 1977, 1990). For the purposes of this analysis, treating Greentown as a field is adopted as an interpretive strategy that foregrounds these internal dynamics and provides a productive framework for understanding how practice is organised and reproduced in this context.

Using Bourdieu’s toolkit, the durability of the Greentown network is explored not simply as a matter of structure or hierarchy, but as a process of social practice sustained through habitus, capital and doxa understood as the taken-for-granted conditions of the field, through which symbolic power and symbolic violence shape practice, secure compliance and insulate the network from external challenge (Bourdieu & Wacquant, 1992; Loyal & Quilley, 2017). Within this space, the struggle for dominance can be read as taking place between the State’s formal governance structures and the criminal network’s informal governance system headed by A2. The result is an overlapping system of governance that appears to shape behaviour within the field and to normalise certain practices for the residents of Greentown. Thus, when Redmond (Redmond, 2015, p. 129) describes the residents who “stoically go about their business… keeping their heads down”, this can be interpreted as illustrating how residents may have developed a habitus oriented towards caution, avoidance and acceptance of prevailing field conditions. Under such conditions, compliance is often secured without the need for overt coercion, contributing to the relative stabilisation of the network’s authority whilst avoidance patterns appear to reinforce territorial control and limit residents’ engagement with the state (Loyal & Quilley, 2017). Residents can, therefore, be understood as having accepted the informal governance structures as a way of navigating the field conditions and understand the ‘rules of the game’ (Bourdieu, 1990; Bourdieu & Wacquant, 1992).

Young people exposed to the same structural conditions as residents may develop divergent dispositions, and their engagement with the network varies; some participate apparently voluntarily and embrace the opportunities on offer whilst others are coerced by older members of the network into engaging. Theirs is often not the disposition of avoidance common among residents but one that is more action-oriented. In these engagements, young people look, however misinformed, to boost their capital (social, economic or cultural) as part of a strategy oriented towards improving their field position by adopting behaviours and actions that favour the forms of capital most valued in the field (Bourdieu, 1986). By becoming part of the network, young people are reported as mimicking the behaviours of established members and in doing so contribute to the reproduction of the field conditions and the maintenance of the locus of physical and symbolic dominance exerted by A2. In turn, this can contribute to the reproduction of the field conditions, rendering criminalised behaviour more readily understood as normal, strengthening the informal governance structures and extending the reproduction of the network across generations (Bourdieu, 2001). As noted by Redmond:

“many [young people] have also experienced harsh and abusive child rearing… with siblings and parents also involved in criminality”

(Redmond, 2015, pp. 137–138).

These conditions can be interpreted as amounting to socialisation into instability and violence, shaping dispositions that resonate with the network’s norms and orienting participation towards the routine rather than the exceptional (Burawoy, 2019; Storm-Mathisen, 2025).

In Greentown, the network can be interpreted as a family-centered criminal governance order run both practically and symbolically by A2, and his authority can be read through a Bourdieusian lens as resting on accumulated symbolic capital, generated through reputation, narrative and perceived capacity for sanction. This capital underpins his symbolic power, enabling him to shape expectations and regulate behaviour within the Greentown network.

The aim of the day-to-day running of the network appears to be the maintenance of the field conditions that sustain the habitus of the network. For this group, maintaining the status quo of the kin-based governance system is, in a Bourdieusian sense, read as a struggle to maintain a field that supports and rewards particular positions and practices (Bourdieu, 1999).

For associates, compliance is shaped less by trust or kinship than by a practical, embodied sense of the network’s doxa, the taken-for-granted “rules of the game” that organise practice within the Greentown field (Bourdieu, 1989). Their position outside the dominant family places them in what the study describes as a “principal–agent type governance” structure (Redmond, 2015, p. 119), where the family’s expectations and the associates’ vulnerabilities combine to structure behaviour. These agents are drawn into dependent relations with A2’s network, reinforced by drug debts and moneylending, which create “push forces associated with debt or obligation” (Redmond, 2015, p. 156) and bind them into forms of client-like dependency. Repayment is extracted either through direct financial return or through the commission of criminal acts, resulting in a coercive form of indebtedness that functions as an informal mechanism of control within the field (Redmond, 2015, p. 122).

Unlike family members, whose position in the hierarchy is secured through trust and intergenerational socialisation, associates are kept in line through a mix of symbolic and material constraints, underpinned by A2’s capacity for sanction. As one interviewee put it:

“people are so afraid of him… they know if they mess with him there’s going to be some kind of consequence”

(Interview 009, Redmond, 2015, p. 98).

This ever-present possibility of violence, supported by “perceptions, stories and myths about A2 and his family” (Redmond, 2015, p. 123), can be understood as contributing to conformity that becomes both habitual and pragmatic within the logic of the field (Loyal & Quilley, 2017). While family members actively accumulate and reproduce symbolic capital, associates encounter its effects primarily through symbolic power, and residents are more likely to experience its internalisation as symbolic violence, expressed in patterns of cautious compliance and avoidance.

This coercive, obligation-based mode of regulation stands in contrast to the forms of trust, loyalty and inherited privilege that govern relationships within A2’s family group, where compliance appears to arise less from fear than from a shared habitus cultivated across generations. In short, this forms “a multi-generational family-based criminal organisation” that operates through “well-embedded institutional rules and routines” (Redmond, 2015, p. 137). These kinship bonds help generate a shared habitus, inculcated from birth, which provides family members with the dispositions needed to intuitively navigate the network’s doxa and to enact the self-governing behaviours that A2 expects. Membership of “the most feared family in Greentown” (Redmond, 2015, p. 126) brings elevated privilege, grounded in “trust” and in a familial socialisation that inculcates the Greentown field as a naturalised order, one in which the family rules. This intergenerational succession is visible in the cultivation of B2 and D2, who are groomed as successors and who already carry “authority and intimidation rights… well beyond their actual age and maturity” (Redmond, 2015, p. 130). The unity of this familial habitus depends in significant part on the symbolic and practical authority of A2, which positions him as the central organising force of the field. His presence anchors the family’s dominance and provides the symbolic capital through which they appear able to sustain a self-reinforcing governance regime that remains largely immune to external challenge. However, the symbolic capital that the family accumulates does not ensure residents’ compliance but enables and supports the use of symbolic power through which expectations are set and reinforced.

In short, the mechanisms of compliance are increasingly internalised, making the criminal hierarchy appear natural and, in turn, contributing to the reproduction of the field conditions present in Greentown. So long as these conditions remain in place, the reproduction of the conditions is likely to persist, and the family is likely to remain the dominant force within the field.

While habitus explains how practices become embodied and normalised within the Greentown field, it is through the mobilisation and distribution of capital that A2 and his family can be understood as actively working to reproduce these field conditions and consolidate their authority (Bourdieu, 1986). The study consistently highlights that the network operates as a “multi-generational family-based criminal organisation” (Redmond, 2015, p. 95) that acquires and deploys various forms of capital—economic, social, cultural and, critically, symbolic—in ways that sustain dominance and regulate others. The close family ties forming the inner core of the network rely on social capital, grounded in trust and the “well-embedded institutional rules and routines” that structure relationships within the kinship group (Redmond, 2015, p. 137). Practices such as “being seen in the car with A2, D1” (Redmond, 2015, p. 124) generate social capital by association (Bourdieu, 1986/2011), functioning as patronage mechanisms that enhance the perceived status of those involved and illustrating how ‘capital by association’ is accumulated and converted within the logic of the field.

The Greentown study also draws attention to the importance of cultural capital, particularly in its embodied form, which is acquired through early socialisation within the family and through repeated exposure to the routines of offending. Family members hold a distinctive set of practical skills and behavioural competencies that allow them to manage risk, navigate encounters with the police and read the shifting expectations of the network. Notably, A2’s ability to appear calm, courteous and cooperative during searches, while simultaneously retaining control of the situation (Redmond, 2015, p. 140), illustrates this embodied cultural capital and the way it can provide strategic advantage within the field.

D1 offers a further example of how cultural capital operates in practice:

“D1… has saleable skills, not least his driving proficiency”

(Redmond, 2015, p. 122)

Although not part of the core family, he has acquired certain practical skills that are valued within the network, most notably his driving proficiency. These skills form a limited but meaningful form of cultural capital that elevates his status relative to other associates and allows him to function as an effective intermediary for senior agents (Redmond, 2015, p. 122). However, because this cultural capital is not accompanied by the deeper embodied dispositions held by family members, D1’s capacity for self-management appears uneven, and he remains subject to punishment when he acts impulsively or without discipline (Redmond, 2015, p. 102). This contrast helps reinforce the internal hierarchy of the network: family members possess a more fully developed cultural capital that supports trust, discretion and symbolic authority, while associates like D1 hold narrower, task-specific skills that offer utility but do not grant access to the family’s inner domain or its accumulated symbolic power.

Moneylending and the accumulation of drug debt act as governance mechanisms but also operate as forms of economic capital, revealing the routine transfer of this capital between agents and the dependence it creates, as noted in this quote:

“…levers debtors, often women, who had never been involved in crime, to become involved in theft and shop lifting activity to service the debt”

(Redmond, 2015, p. 122).

Economic capital, expressed through debt-based dependency and delegated labour, enables senior agents to extract value and sustain coercive governance while often insulating themselves from direct exposure to police scrutiny. These practices reflect the broader Bourdieusian dynamic in which economic, social and cultural capital are mobilised and converted to help reproduce the field’s conditions, consolidating the symbolic power of A2, whose “perceptions, stories and myths” contribute to maintaining his status and encourage self-regulation among associates and residents (Redmond, 2015, p. 123).

The “perceptions, stories and myths” surrounding A2 are central to the construction of his status and provide him with an “untouchable mythology… in the minds of network clients and associates” (Redmond, 2015, p. 159). Mythmaking sustains A2’s symbolic dominance, positioning him beyond ordinary challenge while enabling other members of the wider family to accumulate a similar standing. The mythology that surrounds the family as a whole can be understood as a form of symbolic capital, which underpins their authority by transforming fear, reputation and a sense of ambiguous respectability into a powerful source of legitimacy that reduces reliance on overt violence. As noted here:

“people are so afraid of him… No one would mess with him”

(Interview 009) (Redmond, 2015, p. 98).

This symbolic capital also becomes a vehicle for symbolic violence, a form of power Bourdieu (1991) identifies as the imposition of meaning that subordinates come to accept as natural or inevitable. In Greentown, symbolic violence is evident in the fear generated by A2’s reputation, the anticipation of consequences and the widespread normalisation of the family’s dominance. Over time, these myths appear to become incorporated into the doxa of the field, where assumptions about untouchability, expected compliance and the perceived ineffectiveness of the state are taken for granted. As these beliefs become increasingly naturalised, the network’s authority appears inevitable, making its dominance more difficult to contest in meaningful ways. A2’s mythmaking operates in exactly this way: local stories, rumours and emotionally charged narratives continually regenerate and reinforce his authority within the neighbourhood. Thus, mythmaking contributes to the accumulation of symbolic capital by enhancing A2’s reputation and perceived untouchability. This symbolic capital is then mobilised as symbolic power, shaping expectations and encouraging anticipatory compliance. As these narratives become embedded within everyday understanding, they contribute to symbolic violence, whereby the network’s authority is experienced as natural and inevitable.

Bourdieu’s framework allows Greentown to be interpreted as a field, in an analytical sense, where different forms of capital are exchanged to generate compliance, status and continuity. Habitus shapes everyday dispositions, while doxa makes domination appear normal. Symbolic power, reinforced through myth and managed civility, pushes coercion into the background, and symbolic violence reframes exploitation as opportunity. The network reproduces itself through intergenerational socialisation, economic dependency, spatial ties and limited state influence. Taken cautiously, this suggests that Bourdieu’s approach may also be useful for interpreting other spatial criminal settings organised around family authority, patronage and reputation.

## 7. Can Bourdieu Help?

The discussion that follows is intended as an interpretive and exploratory reading rather than a definitive explanatory account, and the claims advanced should be understood in that context.

This analysis has demonstrated how Bourdieu can be used to help interpret aspects of the knotty problem (Sparrow, 2008, pp. 8–9) presented by the Greentown case. However, for scientists, policymakers and planners, it is whether Bourdieu can offer transferable insights into how networks operate and inform propositions for better managing and controlling them that matters more.

We suggest that the Bourdieusian lens offers a form of necessary fresh thinking regarding powerful neighbourhood-based criminal networks. We argue further that Bourdieu’s theoretical contributions have the capacity to bring together components and mechanisms that have been observed across multiple studies of criminal networks into a cluster of compelling narratives. The value-added properties of narrative lie in their ability to render intelligible fuzzy wicked problems such as Greentown, whereas, perhaps counter-intuitively, precise technical and mechanistic definitions may be better suited to defining tame problems (Rittel & Webber, 1973, p. 160). Bourdieu can be read as offering an analytic vantage point from which to consider how symbolic capital, symbolic power, symbolic violence and symbolic strategy combine to enable effective and efficient (illicit) governance of network subordinates and neighbourhood stoics. Bourdieu can help illuminate why individual agents may appear to exercise discretion in reasonably predictable ways, developing responses that are strategic in a practical sense to the threats and opportunities presented by a capricious network context.

In closer focus, a Bourdieusian reading helps explain how A2 appears able to govern Greentown so efficiently and why the network has endured for so long without repeated recourse to enacted violence. Stories of what might happen if one does not comply, based on supposed historical and contemporary accounts, are stitched into the fabric of the Greentown narrative. The network’s ability to routinely restock the supply of street sellers (children) when they are lost to law-enforcement intervention contributes, at least from the perspective of residents, to a sense that this state is natural and enduring (Sparrow, 2008, pp. 231–241). In sum, the myths surrounding A2 may pose as formidable a challenge to reducing the network’s influence as the network’s actual capabilities.

Our critical observation of the main corpus of work regarding young people’s involvement in serious crime and criminal networks is that while social network analysis often succeeds in isolating relational structures, strategies to understand networks can be read as remaining partial. They are commonly under-the-bonnet treatments, focusing on nodes (people), transactions (links or edges) and the interplay between them, and less on the story of the network as a whole. We argue that, in addition to understanding moving parts, networks may benefit from being considered analytically as entities in their own right. For example, networks operating in legitimate markets demonstrate resilience to external shocks by encouraging individuals to act outside immediate self-interest and to cooperate, at least in the short term. Shared interests reduce coordination costs by specifying tacit rules of behaviour that are widely shared (Pilbeam et al., 2012). It is the potential for certain iterations of networks to solidify such tacit rules that generates institutional-like properties, creating stable expectations of behaviour (Hodgson, 2006, p. 2) and default norms, marking them out as both highly flexible yet potentially internally coercive, particularly where dominant hierarchies are integral to their structure.

The criminal network literature has advanced understandings of network structure (Morselli, 2009), resultant properties (McGloin & Piquero, 2010; McGloin & Nguyen, 2013), why and how young people join networks (Weisburd et al., 2020), why they are retained (Joseph, 2024; Tollin et al., 2025), how they indicate their motivation to leave (J. A. Densley & Pyrooz, 2017) and how they exit (Pyrooz & Decker, 2011; O’Brien et al., 2013). Network governance has also been examined as a trade-off between efficiency and security (Morselli, 2010). Network structures and internal alliances vary by illicit activity type (Breuer & Varese, 2023) and functional role (Malm & Bichler, 2011). However, perceptions of whether networks are structured organisations or loose alliances diverge, with ethnographic accounts often emphasising informality, while professional observers are more likely to see formal structure (Windle & Briggs, 2015, p. 1180). Links between network actors have also received attention for their fluidity (Bouchard, 2020), including distinctions between substantive and instrumental relationships (Campana, 2016).

Where conceptual ideas of network properties have been advanced, including capital, this work has tended to focus on assets assigned to agents or groups and their contributions to sustainability. Bright and Whelan (2020) identify the importance of capital for network strength, highlighting actors with high social capital—those with numerous or strategically positioned connections (p. 119)—and human capital, including leadership, specialised skills or exclusive access to resources (p. 121). Such distinctions help to identify vulnerabilities and opportunities for disruption (Sparrow, 2008, pp. 27–28). However, whether disruption is best achieved through targeting actors with elevated capital, leadership figures or peripheral agents remains context-contingent and contested (Redmond, 2015, p. 32).

In youth justice, conventional interventions first turn to Evidence-Based Programmes (EBP) (Drake et al., 2009) to assure effectiveness and value for money. Yet repeated systematic reviews of evidence regarding child and youth violence (Youth Endowment Fund, 2021; Walsh et al., 2025), youth involvement in gangs and organised crime (Mellgren et al., 2026; Adamse et al., 2024) and drug-related intimidation (Murphy et al., 2017) identify few programmes meeting high confidence thresholds. For those that do, confidence in their effectiveness remains low to moderate and limited in scope. These findings could be seen as lending support to Rittel and Webber’s claim that social scientists repeatedly refine solutions to tame problems rather than design responses to wicked ones (Rittel & Webber, 1973). Programmes such as Multisystemic Therapy (MST) and Functional Family Therapy (FFT, including FFT-G) frequently emerge as frontrunners, yet Greentown’s own experience with FFT shows mixed results. While effective with therapy-ready families, it proved insufficient where families were traumatised into distrust by network coercion (Mulcahy et al., 2024, p. 239). Even if fully effective by programme criteria, improvements in family functioning may represent only a limited response when coercion originates outside the family system.

Importantly, certain networks’ capacity to bestow or withdraw capital through governance has received comparatively less attention. Below, we return to Bourdieu to explore what his theory may contribute to addressing this gap. Drawing on Greentown, we focus on mythology as an overarching governance phenomenon that enables the network to encourage acceptable behaviour among trusted members, constrain opportunism among associates and bound permissible practices, cultivating self-governance among residents who stoically go about their daily lives.

## 8. Busting Myths?

What if myth busting became a complementary and sustained non-kinetic (Everton, 2012) focus for a collective of state agents seeking to tame a network, alongside the ‘simultaneous application of prevention, deterrence and rehabilitation “best practices”’ (J. Densley, 2011, p. 9)? ‘Pulling levers’ as a strategy to rescue individuals is not new (Braga & Weisburd, 2012) and has more recently demonstrated that it can contribute to deterring network-related crime, building public trust in policing authorities and doing so without the usual concerns about displacement (Braga et al., 2026). However, thinking creatively about pulling levers to challenge or unsettle a malign truth that governs the lives of citizens in situations like Greentown opens up another potentially powerful tool for further consideration and deliberation. Such models of intervention resonate with recent reflections from behavioural scientists who see strategic value in shifting from individual to system-level nudges (Hallsworth, 2023). The value of portfolio-based approaches ‘that are complementary and can shift complex systems by focusing on multiple intervention points at a given time’ has also been noted by the United Nations (Wellsch, 2022).

What would it look like if strategic efforts were focused on undermining the supposed truths and enabling conditions that permitted A2’s mythology to persist over many years, by identifying potential points of symbolic or narrative sabotage (Sparrow, 2008, pp. 27–29)? Framing the problem in this way invites different questions and opens space for alternative approaches involving non-justice disciplines and creative capacities, rather than focusing exclusively on senior figures, foot-soldiers or other capital-rich individuals. Importantly, such soft strategies are not limited to tame problems. Roberts and Everton (2011), writing on dark networks involving criminality and terrorism, suggest that non-kinetic strategies that are more subtle and the patient application of power are necessary to accompany more overt kinetic interventions (p. 4). In addition to strengthening criminal sanctions and welfare support, promising strategies to combat domestic violence and sexual and gender-based violence now include public awareness campaigns aimed at ‘challenging existing myths, misconceptions and established beliefs’ (Eolas Magazine, 2022). These strategies operate at a distance from direct engagement, seeking instead to enhance victims’ and survivors’ agency.

We do not claim to offer ready answers to the problems outlined in Greentown. In fact, our suggestion is that, as with any other theory-led idea proposed for a complex and dangerous environment, our assumptions should be remotely road tested by front-line professionals with tacit and craft knowledge of the terrain. This would safely test the core assumptions and may result in the development of prototypes and safe-to-fail experiments. This process is analogous to the process that led to the development of the Greentown model itself. Detailed problem examination represents a necessary first step in addressing wicked problems. Too often, solutions to wicked problems are offered prematurely (Rittel & Webber, 1973, p. 161) and take the form of conventional ‘tame problem’ remedies with insufficient potency or bandwidth. Our contribution, therefore, is deliberatively exploratory. It suggests that theory, informed by Bourdieu’s conception of practice, may help generate new ways of thinking and potential strategies worthy of further deliberation in response to a policy problem with which many jurisdictions continue to struggle.

## 9. Limitations

We are conscious that the substance of our examination is a single case, Greentown, and that any more general inferences must be treated with caution. However, the Greentown methodology has been replicated four times in Ireland in carefully (purposive) sampled locations, allowing for some wider theoretical representativity. It is also important to recognise that the data sourced for the Greentown study derives from second-hand observations from police officers, as opposed to stories and accounts secured directly from children and families. The Twinsight method was designed deliberately as a non-invasive strategy to respond to perceived risks of harm for individuals cooperating with the study. Anonymised locations, anonymised respondents and anonymised research subjects, assured through Twinsight, were intended to respond proportionately to this risk. Assuming that, in these circumstances, Twinsight is considered to have merit, there would be value in broadening the disciplinary base of respondents to include other professions who work directly with the children who are the focus of this analysis. Police officers were the first choice of respondents, partly reflecting limited resources, but this decision also aligns with Malm’s observation that, in the absence of abundant resources, ‘law enforcement intelligence sources are sufficient to identify robust network descriptions’ (Malm & Bichler, 2011, p. 291).

Bourdieu’s Theory of Practice offers a powerful lens for interpreting how symbolic power, myth and embodied dispositions shape criminal network governance. However, it is not the only lens that could be applied to understanding the role of mythology in criminology. For example, cultural criminology addresses mythology, narrative and meaning making within criminal subcultures, although it tends not to foreground structured relational dynamics in the way Bourdieusian analysis does. Accordingly, Bourdieu’s theory is not presented as the only viable analytical option, but as one among several possible theoretical approaches to examining the phenomenon at hand.

While our argument is necessarily tentative and may attract criticism for not conforming to conventional rules of scientific evidence, we intentionally advance a rationale for fresh thinking that approaches the problem as a whole, rather than as a constellation of self-contained battlegrounds where individual outcomes are imagined in isolation. Programme-level gains and effect sizes, while valuable in bounded contexts, are unlikely on their own to address the scale and complexity of the adversities facing young people and their families in situations such as Greentown.

The paper proposes that myth busting and symbolic disruption may represent promising avenues for further exploration in relation to disrupting criminal networks. However, these ideas remain speculative, grounded in theoretical analysis, and no concrete strategies, delivery mechanisms or implementation risks are set out, nor does the paper examine potential ethical issues or unintended consequences that might arise from myth-busting strategies. While the paper considers the analytical implications of myth busting, its practical application is not developed and remains exploratory.

Overall, these limitations do not undermine the contribution of the paper but highlight that it should be read as theory-informed, evidence-aware analysis and problem framing, rather than as definitive causal inference. In writing the paper, the authors sought to foreground the complexity of the problem and to argue that programme-level effect sizes alone are insufficient to meet the realities of wicked problems such as those illustrated by Greentown.

## Data Availability

No new data were created or analysed in this study.

## Notes

1 European Crime Prevention Award 2020 [Downloaded 18 February 2026 @ 07:20 https://eucpn.org/sites/default/files/document/files/2011_Toolbox_Family-based%20crime_LR.pdf, accessed on 25 February 2026.

2 Higher Education Authority 2023—Greentown awarded €1.3 million for research impact [Downloaded 18 February 2026 @ 07:38] University of Limerick 2023|Funding, Governance and Performance, Higher Education Authority.

3 Department of Justice press release ‘Minister McEntee and Minister Browne welcome commencement of legislation targeting those who groom children to commit crime 24/0524 [downloaded 18 February 2026 @07:46 Minister McEntee and Minister Browne welcome commencement of legislation targeting those who groom children to commit crime.

4 The sole use of police respondents was a deliberate methodological strategy intended to trade off reasonably informed and proximal accounts of criminal network activity against perceived safety issues for children, families and community members living in the Greentown neighbourhood. Impartiality was not assumed. Opinion-only police perspectives were mitigated by active use of Twinsight to internally triangulate police accounts about specific people and specific events at specific times. The reliance on one data source and specifically police respondents is highlighted in the limitations for the study, in terms of second rather than first-hand accounts, while recognising the valuable vantage point of police officers as experienced nested observers.
